# Angiotensin II-Type I Receptor Antagonism Does Not Influence the Chemoreceptor Reflex or Hypoxia-Induced Central Sleep Apnea in Men

**DOI:** 10.3389/fnins.2020.00382

**Published:** 2020-04-28

**Authors:** Courtney V. Brown, Lindsey M. Boulet, Tyler D. Vermeulen, Scott A. Sands, Richard J. A. Wilson, Najib T. Ayas, John S. Floras, Glen E. Foster

**Affiliations:** ^1^Centre for Heart, Lung, and Vascular Health, School of Health and Exercise Science, University of British Columbia – Okanagan, Kelowna, BC, Canada; ^2^Division of Sleep and Circadian Disorders, Brigham and Women’s Hospital and Harvard Medical School, Boston, MA, United States; ^3^Department of Physiology and Pharmacology, Hotchkiss Brain Institute, Cumming School of Medicine, University of Calgary, Calgary, AB, Canada; ^4^Sleep Disorders Program, University of British Columbia, Vancouver, BC, Canada; ^5^Respiratory and Critical Care Divisions, University of British Columbia, Vancouver, BC, Canada; ^6^University Health Network and Sinai Health System Division of Cardiology, Department of Medicine, University of Toronto, Toronto, ON, Canada

**Keywords:** chemoreceptor reflex, angiotensin receptor, hypoxia, sleep apnea, human

## Abstract

Components of the renin-angiotensin system (RAS) situated within the carotid body or central nervous system may promote hypoxia-induced chemoreceptor reflex sensitization or central sleep apnea (CSA). We determined if losartan, an angiotensin-II type-I receptor (AT_1_R) antagonist, would attenuate chemoreceptor reflex sensitivity before or after 8 h of nocturnal hypoxia, and consequently CSA severity. In a double-blind, randomized, placebo-controlled, crossover protocol, 14 men (age: 25 ± 2 years; BMI: 24.6 ± 1.1 kg/m^2^; means ± SEM) ingested 3 doses of either losartan (50 mg) or placebo every 8 h. Chemoreceptor reflex sensitivity was assessed during hypoxic and hyperoxic hypercapnic ventilatory response (HCVR) tests and during six-20s hypoxic apneas before and after 8 h of sleep in normobaric hypoxia (F_*I*_O_2_ = 0.135). Loop gain was assessed from a ventilatory control model fitted to the ventilatory pattern of CSA recorded during polysomnography. Prior to nocturnal hypoxia, losartan had no effect on either the hyperoxic (losartan: 3.6 ± 1.1, placebo: 4.0 ± 0.6 l/min/mmHg; *P* = 0.9) or hypoxic HCVR (losartan: 5.3 ± 1.4, placebo: 5.7 ± 0.68 l/min/mmHg; *P* = 1.0). Likewise, losartan did not influence either the hyperoxic (losartan: 4.2 ± 1.3, placebo: 3.8 ± 1.1 l/min/mmHg; *P* = 0.5) or hypoxic HCVR (losartan: 6.6 ± 1.8, placebo: 6.3 ± 1.5 l/min/mmHg; *P* = 0.9) after nocturnal hypoxia. Cardiorespiratory responses to apnea and participants’ apnea hypopnea indexes during placebo and losartan were similar (73 ± 15 vs. 75 ± 14 events/h; *P* = 0.9). Loop gain, which correlated with CSA severity (*r* = 0.94, *P* < 0.001), was similar between treatments. In summary, in young healthy men, hypoxia-induced CSA severity is strongly associated with loop gain, but the AT_1_R does not modulate chemoreceptor reflex sensitivity before or after 8 h of nocturnal hypoxia.

## Introduction

Angiotensin-II (ANG-II) is a physiologically active hormone of the renin-angiotensin system (RAS) and has target receptors in various tissues, including the carotid bodies and central nervous system (Allen, 1998; Li et al., 2006). Animal models have demonstrated that ANG-II primarily exerts its effect on the carotid body through the ANG-II type-I receptor (AT_1_R) (Allen, 1998). AT_1_Rs are found on chemosensitive glomus cells of mouse carotid bodies and are upregulated following ANG-II infusion (Allen, 1998). Administration of ANG-II consequently elevates peripheral chemoreceptor activity and augments the chemoreceptor response to hypoxia in animal models of heart failure (Allen, 1998; Li et al., 2006). In isolated rat carotid bodies, blockade of the AT_1_R with losartan, severely reduces the afferent response to hypoxic-hypercapnic challenges (Roy et al., 2018), suggesting the AT_1_R is critical for peripheral chemoreceptor sensitivity.

Exposure to sustained hypoxia also sensitizes the chemoreceptor reflex response in both animals and humans (Aaron and Powell, 1993; Richard et al., 2014). In rats exposed to chronic hypoxia, there is increased expression of angiotensinogen and AT_1_Rs in carotid body glomus cells, and activation by ANG-II is associated with increased excitatory response which is attenuated by AT_1_R blockade (Leung et al., 2000; Lam and Leung, 2003). In rats, administering an AT_1_R blocker attenuates the long-lasting activity in the carotid sinus nerve (CSN) following acute hypoxic-hypercapnic bouts, demonstrating a critical role for the AT_1_R in the chemoreceptor reflex response to intermittent hypercapnic hypoxia (Roy et al., 2018). While AT_1_R antagonists (i.e., losartan) effectively mitigate the lasting effects of intermittent hypoxia on muscle sympathetic nerve activity (MSNA) and blood pressure in humans (Foster et al., 2010; Jouett et al., 2017), it remains to be determined if AT_1_R blockade attenuates chemosensitivity (either peripheral or central) before or after hypoxia in humans.

Heightened chemosensitivity during hypoxia is understood to contribute to the development of periodic breathing with sojourn to high altitude. Mechanistically, the presence of arterial hypoxia acutely increases the ventilatory responsiveness to hypoxia and to hypercapnia (e.g., manifests as a reduced gap between eupneic partial pressure of carbon dioxide (PCO_2_) and the apneic threshold). This heightened peripheral chemoreceptor sensitivity, via an increase in the overall loop gain of the ventilatory control system, leads to reduced stability and overt patterns of cyclic apnea and hyperventilation particularly during sleep. Loop gain – a term that describes the overall stability of the ventilatory control system – combines the unique contributions of chemoreceptor sensitivity (controller gain) as well as how effectively ventilation increases arterial partial pressure of oxygen and reduces the arterial PCO_2_ (plant gain) (White, 2005). While high loop gain is considered the leading mechanism of central sleep apnea (CSA) in patients with heart failure or at altitude, it is also the leading cause of obstructive sleep apnea (OSA) (White, 2005; Edwards et al., 2012). Although acetazolamide has been shown to lower both plant and loop gain, and improves sleep apnea severity (OSA and CSA), no study has pharmacologically lowered loop gain via chemosensitivity and consequently improved sleep apnea (Edwards et al., 2012).

Here we tested whether AT_1_R blockade consequently reduces chemoreceptor sensitivity, loop gain, and the severity of CSA in human subjects. Specifically, we assessed the influence of AT_1_R blockade on chemosensitivity before and after 8 h of nocturnal hypoxia, and on the severity of hypoxia induced CSA as a human model of high-loop gain, chemoreflex-dependent sleep apnea. We hypothesized that losartan administration would attenuate the hypercapnic ventilatory response (HCVR) and the cardiorespiratory response to apnea before and after nocturnal hypoxia. Additionally, we hypothesized that losartan would reduce the severity of hypoxia induced CSA by reducing loop gain during 8 h of nocturnal hypoxia.

## Materials and Methods

### Ethical Approval

This study was approved by the University of British Columbia Clinical Research Ethics Board (H17-02920), was registered as a clinical trial (ClinicalTrials.gov; NCT03335904) and conformed to the latest revision of the Declaration of Helsinki. Prior to enrollment in the study all participants provided written informed consent.

### Participants

Male participants (*n* = 14) recruited from the University of British Columbia – Okanagan campus were screened to ensure they were normotensive (systolic blood pressure (SBP) < 140 mmHg, diastolic blood pressure (DBP) < 90 mmHg), had normal pulmonary function (>80% of predicted), and were free of sleep disordered breathing (apnea hypopnea index (AHI) < 5 events/hour) based on a home sleep study. Females were excluded from participation because they reportedly develop less severe CSA compared with males at high altitude (Lombardi et al., 2013) and in heart failure (Sin et al., 1999). Additionally, resting ventilation and blood pressure regulation are sensitive to menstrual cycle phase (Behan et al., 2003; Hart and Charkoudian, 2014). Participants were excluded if they have smoked within the past year and have a history of impaired renal function, cardiovascular disease, or respiratory disease. Participants were also excluded if they were taking any medication, prescribed or over the counter, or were obese (BMI > 30 Kg/m^2^).

### Protocol

Participants were asked to abstain from strenuous physical activity, alcohol or caffeine during the 12 h preceding each experimental session. Using a double-blind, placebo controlled, randomized, crossover design participants took either 50 mg of losartan, or a placebo pill three times over the course of 24 h (see section Pharmacological Intervention). Each experimental visit was separated by a 1 week washout period. Losartan was selected to target the AT_1_R as it has a high affinity for the AT_1_R and has been shown to be void of any agonist activity (Ohtawa et al., 1993). The experimental protocol consisted of (1) a hyperoxic hypercapnic ventilatory response (HCVR) test, (2) a hypoxic HCVR test and (3) a hypoxic apnea response (HAR) test measured before and after a night of sleep in a normobaric hypoxia chamber (8850 SUMMIT+; Altitude Tech, Kingston, ON, Canada). In addition, venous blood samples were collected before and after a night of hypoxic sleep and assayed for plasma renin activity (PRA) and aldosterone to confirm effective dosing of losartan. Cardiovascular and respiratory variables were recorded continuously throughout the HCVR and HAR tests. Following the evening pre-tests, participants were instrumented with a sleep system (see section Monitoring Sleep in Normobaric Hypoxia) prior to entering the hypoxic chamber. The fraction of inspired O_2_ within the normobaric chamber was set to 0.135 which has been shown to sufficiently induce CSA in young, healthy men (Lombardi et al., 2013). Eight hours after entering the chamber, participants were wakened, exited the hypoxic chamber and took the third and final dose of their assigned intervention. They then completed the Lake Louise Acute Mountain Sickness (AMS) Scoring System and the ESQ-Cerebral Symptoms Questionnaire (Roach et al., 1993; Beidleman et al., 2007). The chemoreceptor reflex sensitivity tests were repeated 1 h after the last drug or placebo dose.

### Pharmacological Intervention

Participants were randomly assigned to orally ingest either losartan tablets (50 mg), or placebo pills (microcrystalline cellulose, identical in appearance and packaged in identical blister packages) at 8 h intervals. The first of the three doses was taken ∼10 h before the experimental visit. The second dose was given 2 h before the evening chemoreceptor reflex tests while the third dose was given 1 h prior to morning testing. This timing was selected as plasma concentrations of both losartan and E-3174, its active metabolite, peak after a single dose and are not significantly different than concentrations after 7 days of losartan use (Ohtawa et al., 1993). Additionally, we elected the dosing protocol to ensure plasma concentrations of losartan and E-3147 remained sufficiently high throughout the entire protocol. Peak plasma concentration of losartan occurs an hour after ingestion while E-3147 reaches peak concentrations 2 h after ingestion and as such ventilatory response tests were performed within this time frame (Ohtawa et al., 1993).

### Chemoreceptor Reflex Test Instrumentation

All respiratory and cardiovascular parameters were acquired using an analog-to-digital converter (Powerlab/16SP ML 880; AD Instruments, Colorado Springs, CO, United States) interfaced with a personal computer. Commercially available software was used to analyze ventilatory and cardiovascular variables (LabChart V7.1, AD Instruments). During both sets of ventilatory tests, subjects breathed through a mouthpiece while wearing a nose clip, and a two-way non-rebreathing valve. Respired gas pressures were sampled at the mouth and analyzed for the partial pressure of end tidal oxygen and carbon dioxide (P_ET_O_2_ and P_ET_CO_2_ respectively) (ML206; AD Instruments). Expired gases were passed through a 4.7 L mixing chamber (MLA246; AD Instruments), as well as an oxygen (O_2_) and carbon dioxide (CO_2_) gas analyzer connected in series (S-3A and CD-3A, AEI Technologies, Pittsburgh, PA, United States) to measure the fraction of inspired O_2_ and fraction of inspired CO_2_ to calculate resting metabolic parameters including O_2_ and CO_2_ consumption as well as respiratory exchange rate. These parameters were necessary to determine the isometabolic hyperbola. Respiratory flow was also measured near the mouth using a pneumotachograph (HR 800L, Hans Rudolph, Shawnee, KS, United States) and a differential pressure amplifier (PA1 1110, Hans Rudolph). Heart rate (HR) was determined from a standard lead II electrocardiogram (ML132, AD Instruments). Beat-by-beat blood pressure was measured from a cuff placed on the right middle finger using pulse photoplethysmography (Finometer PRO; Finapress Medical Systems, Amsterdam, the Netherlands). A return to flow calibration was completed and the Finometer was referenced to manual blood pressures taken with an automated sphygmomanometer (Carescape V100; GE Medical Systems, Milwaukee, WI, United States) on the contralateral arm three times during baseline of each testing protocol.

### Hypercapnic Ventilatory Response (HCVR)

Two HCVR tests were administered to assess ventilatory and cardiovascular responses to CO_2_, first on the background of hyperoxia and secondly on the background of hypoxia, with the latter maximizing peripheral chemoreflex activity. Each protocol was separated by 10 min. Performing the HCVR test in hyperoxia minimizes the peripheral chemoreceptor contribution to the chemoreflex response, while a background of hypoxia maximizes the peripheral chemoreceptor input (Duffin, 2007). Prior to starting the HCVR tests, participants rested supine for 10 min while breathing through a mouthpiece with their nose-clamped to collect baseline values of all variables. Throughout the test, participants listened to relaxing music with no prominent rhythm. Dynamic end-tidal forcing (see section Dynamic End-Tidal Forcing) was used to clamp P_ET_O_2_ and P_ET_CO_2_ values throughout the HCVR tests (Tymko et al., 2016). In short, P_ET_O_2_ was clamped at 350 mmHg for the hyperoxic HCVR test and at 50 mmHg for the hypoxic HCVR. P_ET_CO_2_ was clamped for 3 min each at 0, +2, +4, and +6 mmHg respective to each subject’s baseline value.

#### Dynamic End-Tidal Forcing

Control of P_ET_O_2_ and P_ET_CO_2_ was accomplished through an end-tidal forcing system which uses independent gas solenoid valves for O_2_, CO_2_, and nitrogen (N_2_) to deliver a precise quantity of each gas into an inspiratory reservoir where it is humidified (Tymko et al., 2015, 2016). Breath-by-breath measures of P_ET_O_2_, P_ET_CO_2_, tidal volume, breathing frequency and minute ventilation were done online using specifically designed software (Labview 13.0, National Instruments, Austin, TX, United States). The forcing system uses P_ET_O_2_, P_ET_CO_2_, inspired and expired tidal volumes feedback and alters the inspired gas to bring end-tidal gas levels to the target value. Feed-forward control of the inspirate is based on estimates of metabolic O_2_ consumption and CO_2_ production and utilizes the alveolar gas equation to determine the required fraction of inspired O_2_ and fraction of inspired CO_2_. Feedback control is accomplished using a proportional and integral error reduction control system.

#### Calculating Chemoreflex Sensitivity

For both hyperoxic and hypoxic HCVR tests minute ventilation was plotted against P_ET_CO_2_ for all participants. Individual data were regressed using a linear mixed effects model (see section Statistical Analyses) from which individual coefficients (i.e., slopes and intercepts) were calculated from random effects. Reported group means were determined from the global model coefficients representing the group’s response to condition and drug.

### Hypoxic Apnea Response (HAR)

Following the HCVR protocols, participants remained instrumented and performed the hypoxic apnea response (HAR) protocol. A 5 min baseline was collected prior to performing six hypoxic apneas during which all respiratory and cardiovascular variables were recorded. Each apneic cycle was repeated 6 times and consisted of (1) 2–3 breaths through a three-way valve connected to a reservoir containing 100% N_2_, (2) a 20 s end-expiratory breath-hold, and (3) 40 s of room air breathing. A nadir arterial oxyhemoglobin saturation (SpO_2_) between 85 and 90% was targeted during each apnea, and the number of N_2_ breaths prior to each apnea was adjusted to achieve this range. Following the hypoxic apnea cycles, all variables were recorded throughout a 5 min recovery period. The HAR test was performed to assess if ventilatory and cardiovascular responses to apnea were influenced by losartan.

### Monitoring Sleep in Normobaric Hypoxia

Sleep disordered breathing was measured using a continuous, overnight, cardiopulmonary monitoring system (Somte PSG, Compumedics, VIC, Australia). The device consists of an oximeter to record SpO_2_, an electrocardiogram to record HR, a pressure transducer to record nasal airflow, chest and abdomen bands to measure respiratory effort, as well as a body position sensor. Additionally, monitoring of FP1-A2 and FP2-A1 by electroencephalography was combined with electrooculography of the left and right eye to assess sleep vs. wakefulness. Total sleep time was produced once wakefulness and sleep had been scored. The data were manually scored by the same investigator (Profusion 4, Compumedics, VIC, Australia) for the calculation of the AHI, the oxygen desaturation index (ODI) and the peak and nadir oxyhemoglobin saturation according to the criteria established by the American Academy of Sleep Medicine (Berry et al., 2012). In short, apneas (≥90% reduction in peak nasal pressure) and hypopneas (≥30% reduction in peak nasal pressure) were required to be at least 10 s in duration. Additionally, hypopneas had to be associated with a ≥4% desaturation. Arousals and sleep staging were not scored.

### Dynamic Loop Gain During Sleep

Loop gain is the dynamic ventilatory drive response that occurs consequent to a reduction in ventilation. We modified an established method to estimate loop gain during sleep (Terrill et al., 2015). The method involved (1) extracting scored respiratory events, and (2) generating a ventilation signal (tidal volume × respiratory rate, uncalibrated) based on the overnight airflow signal (nasal pressure, linearized). For each window of data (3 min, modified from the usual 7 min to fit the faster cycling of hypoxia-induced CSA), ventilation data were used to model (i.e., explain) future values of ventilatory drive, where ventilatory drive was considered to be equal to ventilation between events (during central apneas ventilatory drive was considered to be subthreshold; i.e., late in the apnea, chemical drive increases and passes zero at the point of central apnea cessation). Using this approach, the average dynamic loop gain was calculated (for each participant, on each experimental night) by taking the median of the values from each 3 min window.

### Venous Blood Sample Collection and Processing

Venous blood samples taken for the measurement of PRA and aldosterone were drawn once in the evening following the ventilatory response tests and once in the morning at least 2 h following administration of the prescribed drug. With oral administration of losartan, PRA levels increase (Goldberg et al., 1993) which we utilized to confirm functional blockade of the AT_1_R. Venous blood samples were collected in two 6.0 mL EDTA coated vacutainers. Collected samples were centrifuged at 4°C and separated into 2.0 mL aliquots. The separated plasma was stored in flat top microcentrifuge tubes and stored at −80°C until later analyzed. PRA and aldosterone were assayed by radioimmunoassay techniques and the PRA assessed by measuring the amounts of ANG-I generated per hour.

### Statistical Analyses

Drug treatment was assigned using an online randomization tool^1^. All statistical analyses were performed in R statistical language (R Foundation for Statistical Computing, Vienna, Austria), lme4 (Bates et al., 2015), lmerTest (Kuznetsova et al., 2017), and emmeans (Lenth, 2019) statistical packages. Mixed effect linear modeling was used to interrogate all defined relationships and the correlation between loop gain and CSA severity, but not AMS responses. Across all models, participant was considered as a random effect, allowing for variable intercepts for each participant. When a significant F-test was achieved, pairwise comparisons were made with a Tukey’s *post hoc* analysis to determine differences between the least square means. Statistical significance was set at a level of *P* < 0.05. A Mann–Whitney *U*-test was run on the AMS scores to detect differences in responses based on drug. With the exception of AMS scores and ESQ-Cerebral Symptoms Questionnaire (presented as medians and interquartile range [IQR]) data are presented as means ± SEM.

## Results

### Participants

**FIGURE 1:**
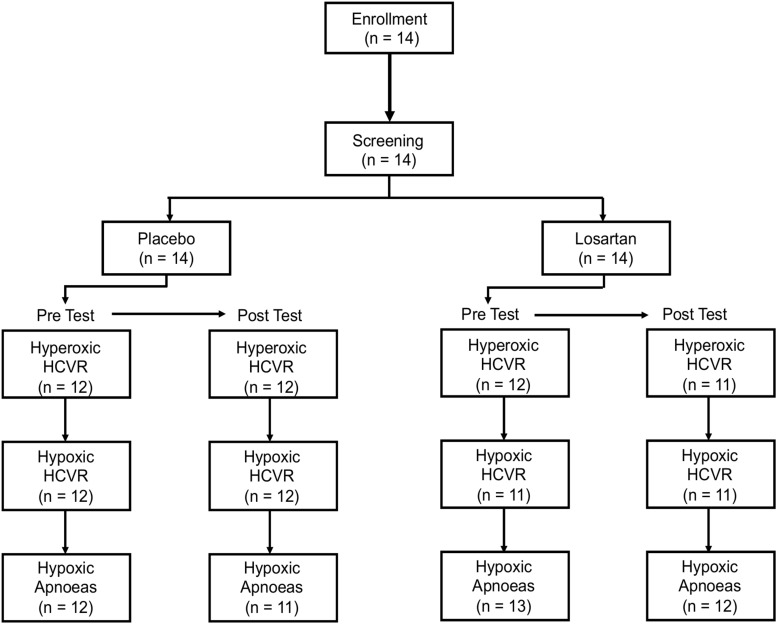
Participant flow chart.

Due to technical and logistical limitations, three HCVR tests were excluded from analysis. An additional subject had less than 2 h of recorded sleep on their experimental night. While taking losartan, two participants developed symptoms of AMS, including nausea and vomiting, causing them to withdraw from further study. Both recovered within a few hours. Despite this, the Lake Louise AMS Score (losartan: 2.5 [3.2] (median [IQR], placebo: 3.5 [3.0]; *P* = 0.3) and ESQ-Cerebral Symptoms Questionnaire (losartan: 3.5 [13.0], placebo: 5.5 [10.2]; *P* = 0.3) did not differ between treatments. Figure 1 illustrates the flow of participants through the chemosensitivity tests and the sample sizes used for statistical analysis.

All participants showed normal pulmonary function [forced vital capacity (FVC) = 105 ± 3% predicted, forced expiratory volume in 1 s (FEV_1_) = 97 ± 3% predicted, FEV_1_/FVC = 93 ± 1% predicted], had a body mass index of 24.6 ± 1.1 kg/m^2^, and were 25 ± 2 years. Participants did not suffer from undiagnosed sleep disordered breathing (AHI = 2.0 ± 0.5, ODI = 0.9 ± 0.3 events/hour), were not taking any additional medications and were normotensive (SBP = 119 ± 2, DBP = 67 ± 2 mmHg).

### Influence of AT_1_R Blockade on Hypoxia-Induced CSA and Loop Gain

**FIGURE 2:**
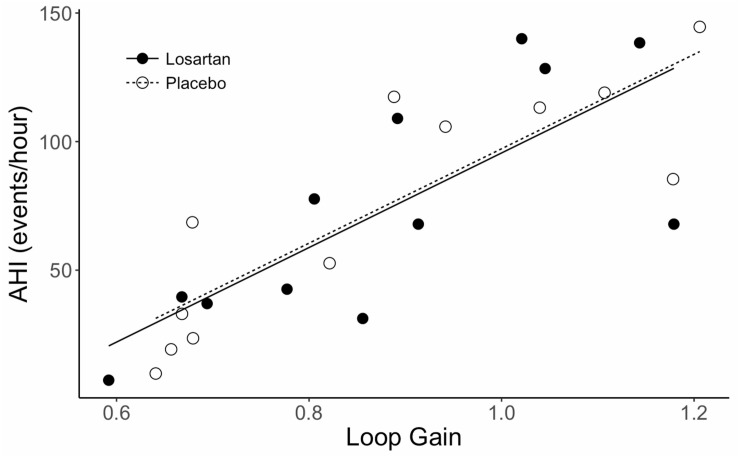
Correlation between AHI and loop gain during sleep in normobaric hypoxia. AHI, apnea hypopnea index. Lines fit to data are based on a mixed effects linear model which accounts for correlation between subjects. The correlation coefficient (*r*) is 0.93, *P* < 0.001.

Sleep time did not differ between losartan and placebo (losartan: 363 ± 26; placebo: 324 ± 16 min, *P* = 0.2). Additionally, we observed similar ODI (losartan: 78 ± 12; placebo: 81 ± 14 events/hour, *P* = 0.7), AHI (losartan: 75 ± 14; placebo: 73 ± 15 events/hour, *P* = 0.9), average oxyhemoglobin desaturation (losartan: 9.5 ± 0.9; placebo: 8.9 ± 1.0%, *P* = 0.3), and nadir SpO_2_ (losartan: 61.3 ± 1.2; placebo: 60.5 ± 1.7%, *P* = 0.7) between losartan and placebo. Loop gain was similar between treatments (losartan: 0.88 ± 0.05, placebo: 0.88 ± 0.06 arbitrary units; *P* = 0.2). There was a strong correlation present between loop gain and AHI (*r* = 0.93, *P* < 0.001; see Figure 2) while loop gain was moderately correlated with ODI (*r* = 0.63, *P* = 0.02).

### Influence of AT_1_R Blockade on Resting Cardiorespiratory Parameters Before and After Normobaric Hypoxia

As anticipated, P_ET_O_2_ was higher following sleep in the hypoxic chamber (90.7 ± 1.1 and 97.3 ± 1.1 mmHg, *P* < 0.001) while P_ET_CO_2_ fell (40.4 ± 0.6 and 36.3 ± 0.6 mmHg, *P* < 0.001). Alveolar ventilation was elevated in the post-test compared with the pre-test (5.4 ± 0.3 and 6.4 ± 0.3 l/min, *P* < 0.05) but was not influenced by drug (losartan: 5.9 ± 0.3, placebo: 6.1 **±** 0.6; *P* = 0.8). Both P_ET_O_2_ (losartan: 93.4 ± 1.0, placebo: 94.6 ± 1.0 mmHg, *P* = 0.03) and P_ET_CO_2_ (losartan: 38.0 ± 0.5, placebo: 38.7 ± 0.5 mmHg, *P* = 0.05) were similar between drug treatments.

Although we observed a drug by condition interaction for SBP (*P* = 0.02), *post hoc* analysis did not reveal any pairwise differences. Losartan did not influence DBP (losartan: 63 ± 1, placebo: 65 ± 1 mmHg), mean arterial pressure (MAP) (losartan: 81 ± 1, placebo: 83 ± 2 mmHg), or HR (losartan: 63 ± 2, placebo: 63 ± 2 bpm; *P* > 0.05 for all comparisons). Similarly, DBP (63 ± 2, post: 65 ± 2 mmHg), MAP (pre: 81 ± 2, post: 83 ± 2 mmHg), and HR (pre: 62 ± 2, post: 63 ± 2) did not differ between the pre- and post-tests (*P* > 0.1 for all conditions).

### Influence of AT_1_R Blockade and Normobaric Hypoxia on the HCVR Test

**FIGURE 3:**
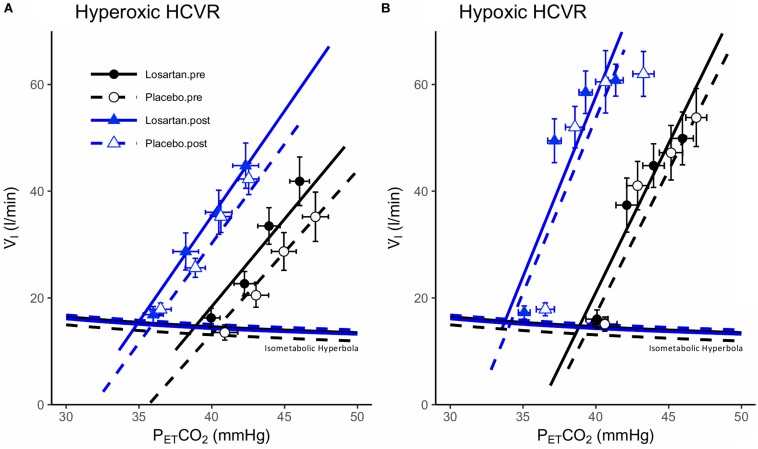
Hypercapnic ventilatory responses during **(A)** hyperoxia and **(B)** hypoxia. Data points are means ± SEM. Lines fit to data using a mixed effects linear model. V̇_*I*_, minute ventilation; P_ET_CO_2_, end-tidal carbon dioxide.

Figure 3 shows mean data, isometabolic hyperbola, and mixed effect linear models illustrating the hyperoxic and hypoxic HCVR before and after 8 h of nocturnal hypoxia for losartan and placebo. As expected, the HCVR was significantly greater in hypoxia (5.3 ± 1.1 l/min/mmHg) compared with hyperoxia (3.6 ± 0.6 l/min/mmHg; *P* = 0.02). There was no significant difference to the HCVR following 8 h of sleep in normobaric hypoxia (5.0 ± 0.5 and 6.2 ± 0.5 l/min/mmHg, *P* = 0.7). Detailed results for these tests are presented in the following two sections.

#### Hyperoxic HCVR Test

The hyperoxic HCVR (Figure 3A) was similar in both losartan and placebo conditions during the pre- (3.9 ± 1.1 and 3.5 ± 0.5 l/min, respectively; *P* = 0.9) and post-tests (3.4 ± 0.5 and 3.6 ± 1.2, respectively; *P* = 0.5), and did not differ following poikilocapnic normobaric hypoxia (*P* = 0.8). Table 1 summarizes cardiorespiratory parameters throughout the hyperoxic HCVR. There was no significant main effect of condition (pre vs. post), or a condition-by-drug-by-P_ET_CO_2_ stage interaction across all cardiorespiratory variables. P_ET_O_2_ and P_ET_CO_2_ were significantly increased from baseline across all stages of the test. A main effect for stage was observed for minute ventilation, which was significantly greater than baseline at all P_ET_CO_2_ stages (see Table 1). MAP was not affected by drug or condition and increased with each P_ET_CO_2_ level (Table 1). The HR response was similar between losartan and placebo and increased with P_ET_CO_2_ level.

**TABLE 1:** Cardiorespiratory parameters measured during the hyperoxic HCVR test.

	Drug	Baseline P_ET_CO_2_		+2 mmHg P_ET_CO_2_		+4 mmHg P_ET_CO_2_		+6 mmHg P_ET_CO_2_	
					
		Pre	Post	Pre	Post	Pre	Post	Pre	Post
V̇_*I*_	Losartan	16 ± 2	17 ± 2	23 ± 2	29 ± 4	34 ± 3	36 ± 4	42 ± 5	45 ± 4
(l/min)	Placebo	14 ± 1	18 ± 1	20 ± 2	26 ± 2	29 ± 3	35 ± 3	35 ± 4	42 ± 3
LSM		18 ± 2		27 ± 2*		37 ± 2*^†^		45 ± 2*^†⁣‡^	
				*Drug: P = 0.8*		***Stage: P < 0.001***		Drug*Stage: P = 1.0	
P_ET_O_2_	Losartan	91 ± 1	96 ± 2	343 ± 7	353 ± 13	337 ± 3	348 ± 15	339 ± 3	347 ± 16
(mmHg)	Placebo	91 ± 1	99 ± 1	339 ± 4	339 ± 7	346 ± 3	346 ± 2	347 ± 3	345 ± 3
LSM		94 ± 3		343 ± 3*		344 ± 3*		344 ± 3*	
				*Drug: P = 0.6*		***Stage P < 0.001***		Drug*Stage: P = 0.3	
P_ET_CO_2_	Losartan	40 ± 1	36 ± 1	42 ± 1	38 ± 1	44 ± 1	41 ± 1	46 ± 1	42 ± 1
(mmHg)	Placebo	41 ± 1	37 ± 1	43 ± 1	39 ± 1	45 ± 1	41 ± 1	47 ± 1	43 ± 1
LSM		38 ± 1		41 ± 1*		43 ± 1*^†^		45 ± 1*^†⁣‡^	
				*Drug: P = 0.09*		***Stage: P < 0.001***		Drug*Stage: P = 1.0	
MAP	Losartan	80 ± 1	81 ± 2	84 ± 2	84 ± 2	87 ± 2	86 ± 2	91 ± 2	89 ± 2
(mmHg)	Placebo	83 ± 2	84 ± 2	86 ± 3	87 ± 2	87 ± 3	88 ± 2	90 ± 3	92 ± 3
LSM		82 ± 1		85 ± 1*		87 ± 1*		91 ± 1*^†⁣‡^	
				*Drug: P = 0.6*		***Stage: P < 0.001***		Drug*Stage: P = 0.9	
HR	Losartan	62 ± 2	64 ± 3	61 ± 2	63 ± 3	64 ± 2	64 ± 3	68 ± 2	70 ± 3
(bpm)	Placebo	63 ± 2	63 ± 2	61 ± 2	60 ± 2	63 ± 2	63 ± 2	66 ± 2	65 ± 2
LSM		63 ± 1		61 ± 1		64 ± 1**^†^**		67 ± 1*^†⁣‡^	
				*Drug: P = 0.4*		***Stage: P < 0.001***		Drug*Stage: P = 0.4	

All values are mean ± SEM. *Indicates a significant difference from baseline (p < 0.05); ^†^significant difference from +2 mmHg (p < 0.05); ^‡^significant difference from +4 mmHg (p < 0.05). LSM, least square marginal means for stage; V̇_*I*_, minute ventilation; P_*ET*_O_2_, end-tidal oxygen; P_*ET*_CO_2_, end-tidal carbon dioxide; MAP, mean arterial pressure; HR, heart rate. Bolded values indicate significant main effects.

#### Hypoxic HCVR Test

The hypoxic HCVR (Figure 3B) was similar in both losartan and placebo conditions during the pre- (losartan: 5.6 ± 1.5, placebo: 5.4 ± 0.6 l/min; *P* = 1.0) and post-tests (losartan: 5.6 ± 1.5, placebo: 6.4 ± 1.2 l/min/mmHg; *P* = 0.9), and did not differ following poikilocapnic normobaric hypoxia (*P* = 0.7). Table 2 summarizes the cardiorespiratory parameters throughout the hypoxic HCVR test. There was no significant main effect of condition (pre vs. post), or a condition-by-drug-by-P_ET_CO_2_ stage interaction across all cardiorespiratory variables. P_ET_O_2_ was slightly lower on losartan (59.7 ± 0.3 and 60.6 ± 0.3 mmHg; *P* = 0.03) and was reduced from baseline across all levels of P_ET_CO_2_ by study design. P_ET_CO_2_ was increased across each stage of the test, but was slightly lower overall on losartan compared with placebo (41.0 ± 0.8 and 42.2 ± 0.8 mmHg; *P* = 0.001). A main effect for stage was observed for minute ventilation, which was significantly greater than baseline at all P_ET_CO_2_ stages (Table 2). Neither MAP nor HR were affected by drug or condition, but both increased with each P_ET_CO_2_ level (Table 2).

**TABLE 2:** Ventilatory parameters measured during the hypoxic HCVR test.

	Drug	Baseline P_ET_CO_2_		+2 mmHg P_ET_CO_2_		+4 mmHg P_ET_CO_2_		+6 mmHg P_ET_CO_2_	
					
		Pre	Post	Pre	Post	Pre	Post	Pre	Post
V̇_*I*_	Losartan	16 ± 2	17 ± 1	37 ± 5	50 ± 4	45 ± 4	59 ± 4	50 ± 5	61 ± 3
(l/min)	Placebo	15 ± 1	18 ± 1	41 ± 5	52 ± 4	47 ± 5	60 ± 5	54 ± 5	62 ± 4
LSM		18 ± 2		47 ± 2*		55 ± 2*^†^		59 ± 3*^†⁣‡^	
				*Drug: P = 0.3*		***Stage: P < 0.001***		Drug*Stage: P = 0.6	
P_ET_O_2_	Losartan	92 ± 2	96 ± 1	49 ± 1	50 ± 0	49 ± 0	50 ± 0	49 ± 1	49 ± 0
(mmHg)	Placebo	92 ± 2	98 ± 1	50 ± 1	51 ± 1	50 ± 1	50 ± 1	50 ± 1	50 ± 1
LSM		92 ± 1		50 ± 1*		50 ± 0*		49 ± 1*	
				***Drug: P = 0.03***		***Stage: P < 0.001***		Drug*Stage: P = 1.0	
P_ET_CO_2_	Losartan	40 ± 1	35 ± 0	42 ± 1	37 ± 1	44 ± 1	39 ± 1	46 ± 1	41 ± 1
(mmHg)	Placebo	41 ± 1	37 ± 1	43 ± 1	39 ± 1	45 ± 1	41 ± 1	47 ± 1	43 ± 1
LSM		39 ± 1		41 ± 1*		43 ± 1*^†^		45 ± 1*^†^	
				***Drug: P = 0.03***		***Stage: P < 0.001***		Drug*Stage: P = 0.6	
MAP	Losartan	82 ± 2	86 ± 3	87 ± 3	94 ± 3	90 ± 3	96 ± 3	91 ± 3	99 ± 4
(mmHg)	Placebo	87 ± 3	88 ± 2	94 ± 3	98 ± 2	98 ± 3	99 ± 3	102 ± 4	101 ± 3
LSM		86 ± 1		94 ± 2*		97 ± 2*		99 ± 2*^†^	
				*Drug: P = 0.3*		***Stage: P< 0.001***		Drug*Stage: P = 0.9	
HR	Losartan	63 ± 2	60 ± 2	77 ± 2	77 ± 2	80 ± 2	82 ± 2	82 ± 2	82 ± 2
(bpm)	Placebo	63 ± 2	60 ± 3	79 ± 2	82 ± 2	82 ± 2	82 ± 2	86 ± 2	88 ± 2
LSM		61 ± 1		78 ± 2*		81 ± 2*		83 ± 2*^†^	
				*Drug: P = 0.7*		***Stage: P < 0.001***		Drug*Stage: P = 0.2	

*All values are mean* ± SEM. *Indicates a significant difference from baseline (p < *0.05); ^†^Significant difference from* +*2 mmHg (p* < *0.05); ^‡^significant difference from* +*4 mmHg (p* < *0.05). LSM, least mean squares; V̇_*I*_, minute ventilation; P_*ET*_O_2_, end-tidal oxygen; P_*ET*_CO_2_, end-tidal carbon dioxide; MAP, mean arterial pressure; HR, heart rate. Bolded values indicate significant main effects.*

### Influence of AT_1_R Blockade and Normobaric Hypoxia on Hypoxic Apnea Response

#### Ventilatory Response

**FIGURE 4:**
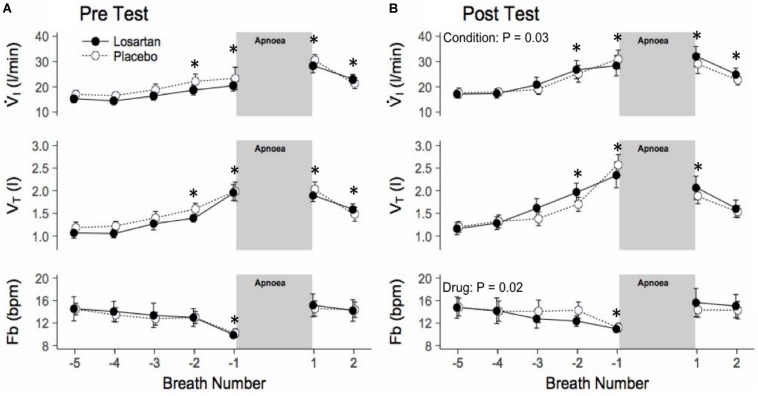
Ensemble averaged breath-by-breath trace of hypoxic apnea response test before **(A)** and after **(B)** normobaric hypoxia. Breath-by-breath trace beginning 5 breaths prior to apnea start. Ventilation is increased prior to the apnea, as participants were asked to take large breaths during nitrogen administration. Values are mean ± SEM. ^∗^*P* < 0.05 compared with respective baseline. V̇_*I*_, minute ventilation; V_*T*_, tidal volume; Fb, breathing frequency.

During the HAR tests SpO_2_ fell to 87 ± 1% and was similar across conditions (*P* = 0.10) and drug (*P* = 0.07). Figure 4 shows the mean breath-by-breath change in ventilatory variables, ensemble averaged across all 6 hypoxic apneas. All ventilatory parameters were significantly elevated from baseline during the first breath following apnea cessation with the exception of breathing frequency which was significantly higher following exposure to normobaric hypoxia (20.9 ± 1.5 and 18.7 ± 1.4 bpm; *P* = 0.03). Losartan attenuated breathing frequency following normobaric hypoxia during the post-test (11.3 ± 1.2 and 12.4 ± 1.2 bpm; *P* = 0.03) but not during the pre-test (12.0 ± 1.1 and 11.9 ± 1.2 bpm; *P* = 0.5). Tidal volume and breathing frequency were similar between conditions.

#### Cardiovascular Response

**FIGURE 5:**
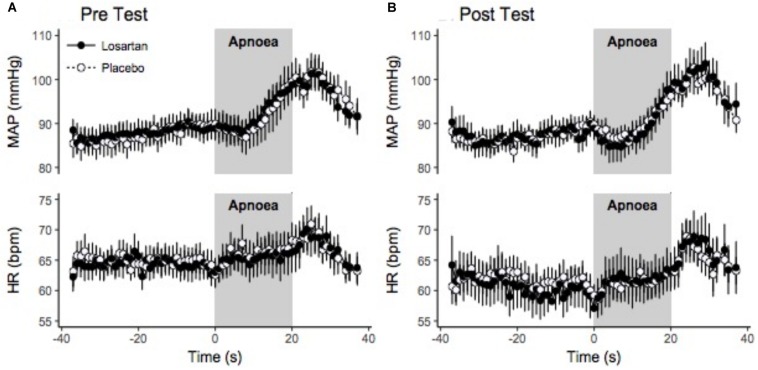
Beat-by-beat ensemble averaged trace of hypoxic apnea response test before **(A)** and after **(B)** normobaric hypoxia. Beat-by-beat trace beginning 30 s prior to apnea start. Values were signal averaged between participants and across the 6 apneas. Values shown are the mean ± SEM. HR, heart rate; MAP, mean arterial pressure.

Figure 5 displays the change in HR and MAP averaged over six apneas. All parameters increased over the duration of the end expiratory breath hold. The 20 s apnea elicited a similar increase in both HR and MAP between both losartan and placebo conditions. Both drug and condition did not have a significant impact on changes in SBP, DBP, or MAP with respect to percent desaturation as summarized in Table 3. There was a trend toward a heightened HR response following sleep in the hypoxic chamber (*P* = 0.06).

**TABLE 3:** Effect of AT1R blockade on cardiovascular sensitivity to hypoxic apnea before (Pre) and after (Post) 8 h of nocturnal hypoxia.

	Drug	Pre	Post	Drug	Condition	Interaction
ΔSBP/ΔSpO_2_	Losartan	3.1 ± 0.6	3.6 ± 0.7	*P* = 0.22	*P* = 0.90	*P* = 0.28
(mmHg/%desaturation)	Placebo	3.1 ± 0.6	2.4 ± 0.6			
ΔDBP/ΔSpO_2_	Losartan	2.3 ± 0.4	2.9 ± 0.5	*P* = 0.60	*P* = 0.30	*P* = 0.23
(mmHg/%desaturation)	Placebo	2.5 ± 0.4	2.2 ± 0.5			
ΔMAP/ΔSpO_2_	Losartan	2.5 ± 0.5	3.1 ± 0.7	*P* = 0.43	*P* = 0.98	*P* = 0.24
(mmHg/%desaturation)	Placebo	2.7 ± 0.5	2.1 ± 0.4			
ΔHR/ΔSpO_2_	Losartan	1.6 ± 0.3	2.5 ± 0.4	*P* = 0.42	*P* = 0.06	*P* = 0.14
(bpm/%desaturation)	Placebo	1.8 ± 0.3	1.9 ± 0.3			

*All values are mean ± SEM. ΔSpO_2_, % of oxyhemoglobin desaturation;ΔSBP, change in systolic blood pressure;ΔDBP, change in diastolic blood pressure; ΔMAP, change in mean arterial pressure;ΔHR, change in heart rate.*

### Influence of AT_1_R Blockade on Markers of the RAS

Angiotensin-I was significantly higher following losartan compared with placebo (8.92 ± 1.32 and 2.94 ± 1.30 ng/mL, *P* < 0.001). Angiotensin-I was also greater following normobaric hypoxia compared with baseline (7.61 ± 1.32 and 4.25 ± 1.30 ng/mL, *P* = 0.02). PRA was elevated by losartan compared with placebo (0.83 ± 0.12 and 0.27 ± 0.12 ng/l/s, *P* < 0.001) and following the sleep in the hypoxic chamber (0.70 ± 0.12 and 0.39 ± 0.12 ng/l/s, respectively, *P* = 0.01). Although aldosterone was higher following normobaric hypoxia compared with baseline (135.8 ± 14.6 and 78.1 ± 14.3 pmol/l, respectively; *P* < 0.01), it was reduced by losartan compared with placebo (76.3 ± 14.6 and 137.5 ± 14.3 pmol/l, *P* < 0.01).

## Discussion

The purpose of this study was to determine if AT_1_R blockade would attenuate (1) the chemoreceptor reflex to CO_2_ and voluntary hypoxic apnea before or after 8 h of nocturnal hypoxia, and (2) the severity of hypoxia-induced CSA through reductions in loop gain. We observed similar ventilatory response to hypercapnia and cardiorespiratory responses to voluntary hypoxic apneas between losartan and placebo before and after nocturnal hypoxia. Additionally, the severity of hypoxia-induced CSA and loop gain was unaffected by AT_1_R blockade. Our data suggests that in healthy, young males the chemoreceptor reflex and the severity of hypoxia-induced CSA occurs through a pathway independent of the AT_1_R and local RAS. Upregulation of the AT_1_R through pathology (e.g., heart failure) or chronic hypoxia may be required before functional changes in the chemoreceptor reflex are observed following AT_1_R blockade.

### Influence of AT_1_R Blockade on the Hypercapnic and Voluntary Hypoxic Apnea Chemoreceptor Reflex Before and Following Nocturnal Hypoxia

The AT_1_R is highly expressed in animal carotid body glomus cells and brain regions involved in cardiorespiratory control including the nucleus tractus solitarius, subfornical organ, median preoptic nucleus, paraventricular nucleus, and rostral ventrolateral medulla (Dampney et al., 2002; Gao et al., 2005; Saxena et al., 2015; Wang et al., 2016; Shell et al., 2019). Activation of AT_1_R by ANG-II in these regions are implicated in the long-term facilitation of the carotid body and the sympathetic nervous system following exposure to intermittent hypoxia (Roy et al., 2018). Additionally, carotid body sensitivity is severely attenuated by losartan in the isolated carotid body preparation (Roy et al., 2018). However, we found the chemoreceptor reflex to hypercapnia and hypoxic hypercapnia to be similar between placebo and losartan which may reflect minimal AT_1_R expression and involvement in chemoreflex modulation in the healthy human. Indeed, our results corroborate those of Foster et al. (2010) who observed no effect of losartan on ventilatory and MAP responses to hypoxia and those of Solaiman et al. (2014) who found no effect of ANG-II infusion in potentiating the ventilatory response to hypoxia or hypercapnia in healthy humans.

Hypoxia activates the carotid body leading to greater ventilatory drive and chemoreceptor reflex sensitivity (Allen, 1998; Marcus et al., 2010). The mechanisms responsible for hypoxic mediated plasticity of the chemoreceptor reflex are not well understood. In rats, carotid body AT_1_R expression was doubled following 4 weeks of chronic hypoxia (10% O_2_) and the carotid body’s sensitivity to ANG-II was enhanced but could be blocked by losartan (Leung et al., 2000). These data support a role of the AT_1_R in mediating carotid body neuroplasticity in response to hypoxia. In addition, carotid body angiotensin converting enzyme activity is doubled following 7 days of hypoxia supporting the presence of a local RAS (Lam et al., 2004). In contrast, we exposed healthy humans to 8 h of continuous hypoxia during sleep which led to minimal chemoreceptor reflex alteration as evidenced by the insignificant change in the hypoxic and hyperoxic HCVR. We observed evidence of augmented chemoreflex drive following 8 h of hypoxia including greater alveolar ventilation, P_ET_O_2_, and reduced P_ET_CO_2_. However, hyperoxic and hypoxic chemoreceptor reflex sensitivity were similar following 8 h of hypoxia and losartan did not modulate chemoreflex sensitivity indicating that AT_1_Rs may not contribute to changes in chemoreflex sensitivity, in healthy young males, following a single night of hypoxia.

Losartan is able to effectively attenuate the increase in sympathetic nerve activity and oxidative stress following both sustained and intermittent hypoxia (Leung et al., 2000; Marcus et al., 2010; Pialoux et al., 2011; Jouett et al., 2017). In rats exposed to 28 days of chronic intermittent hypoxia, the lumbar sympathetic nerve activity response to 20 s apneas was attenuated by losartan (Marcus et al., 2010). The exaggerated lumbar sympathetic and MAP responses to breath hold following chronic intermittent hypoxia were attenuated by losartan, an effect likely mediated by blunted upregulation of AT_1_R expression on the carotid body (Marcus et al., 2010). In contrast, losartan does not influence the ventilatory response to step changes in normocapnic hypoxia in patients with OSA (Morgan et al., 2018). In agreement with our findings, this indicates an alternative mechanism involved in chemoreceptor sensitization that is independent of the AT_1_R. For example, heart failure models suggest a reduction in carotid body blood flow is necessary to reduce neural nitric oxide synthase expression while elevating carotid body AT_1_R expression and ANG-II concentration (Li and Schultz, 2006). Until upregulation of the AT_1_R occurs in animal models of congestive heart failure, losartan has no effect on chemosensitivity (Li et al., 2006). Once AT_1_Rs are upregulated, losartan is able to abolish the ventilatory and renal sympathetic responses to graded levels of hypoxia (Ding et al., 2011). Therefore, the AT_1_R may determine hypoxic chemoreceptor sensitivity in pathological states such as heart failure rather than in healthy humans acclimating to acute hypoxia.

There is limited data regarding chemosensitive signaling mechanisms within the human carotid body. In a previous human study, ANG-II infusion did not potentiate the ventilatory response to hypoxia or hypercapnia further suggesting that the AT_1_R may not play a role in chemosensitivity in healthy subjects (Solaiman et al., 2014). Supporting this, existing data suggests some similarities as well as species differences with respect to carotid body oxygen sensing and reactive oxygen species (ROS) generation. Similar to animal models, the human carotid body releases acetylcholine and ATP in response to hypoxia and expresses haemoxygenase-2, NADPH oxidase (NOX-2), AMP activated protein kinase (AMPK), and oxygen sensitive K^+^ channels (Fagerlund et al., 2010; Mkrtchian et al., 2012; Kåhlin et al., 2014). However, there are also important differences from mice such as the presence of hydrogen sulfide (H_2_S) synthesizing enzyme cystathione-γ-lyase and absence of TASK-3 channels in human carotid bodies (Mkrtchian et al., 2012). Despite these known species differences, there remains no evidence within the literature for the local expression of AT_1_Rs or the components of a RAS in the human carotid body.

### Influence of AT_1_R Blockade on Loop Gain and the Severity of Hypoxia Induced CSA

Elevated loop gain contributes to OSA severity and a high controller gain (mostly measured during wakefulness) is associated with CSA in healthy humans at high altitude, in idiopathic CSA (Ainslie et al., 2013; Terrill et al., 2015), and in patients with heart failure (Solin et al., 2000; Javaheri, 2006). Notably, loop gain during sleep, measured using dynamic application of hypoxic-hypercapnia is remarkably similar to values during wakefulness (Messineo et al., 2018). Thus, individuals with high chemoreflex sensitivity likely suffer from worse CSA severity (Messineo et al., 2018). Our findings support this idea, demonstrating a strong relationship between dynamic loop gain measured during sleep and hypoxia-induced CSA severity (*r* = 0.93). Likewise, interventions that reduce loop gain (acetazolamide, supplemental oxygen) are associated with attenuated central and obstructive sleep apnea severity (Javaheri, 2006; Edwards et al., 2012). In animals, losartan attenuates chemosensitivity, a key contributor to loop gain, which we postulated would reduce CSA severity (Marcus et al., 2010; Ding et al., 2011; Roy et al., 2018). However, we did not observe differences between placebo and losartan for any indices of CSA severity. Similarly, Lombardi et al. (2013) did not find an effect of telmisartan, another AT_1_R antagonist, on the severity of high-altitude CSA during sojourn to 5400 m. This effect is most likely explained by the lack of drug effect on chemosensitivity.

## Limitations and Conclusion

### Limitations

The AT_1_R block may have had a limited effect at baseline due to relatively low RAS activity in our participants. Jones et al. (2007) found that baseline PRA levels are correlated with changes in PRA 24 h following administration of an AT_1_R blockade in healthy volunteers. Participants with the lowest resting PRA levels experienced the smallest increases in PRA following oral administration of the drugs, indicating that AT_1_R blockade has a smaller effect in those that do not have an already upregulated RAS (Jones et al., 2007). However, we found no relationship between changes in PRA and the HCVR. It may be that an upregulated RAS is first necessary for an AT_1_R blockade to have a potent effect on chemosensitivity and sympathetic activity (Li et al., 2006).

A larger dose or dosing period of losartan may have led to different results. At baseline, participants had already received two 50 mg doses of losartan, 10 and 2 h prior to experimentation. We observed significant increases in PRA suggesting functional AT_1_R blockade, although this response was variable amongst participants. Previously, Foster et al. (2010) found that 4 days of losartan (100 mg) blocked the hypertensive response induced by 6 h of intermittent hypoxia. Another human study investigating long-term facilitation of sympathetic nerve activity induced by intermittent hypoxia found that a single 100 mg dose of losartan 1 h prior to experimentation was sufficient to abolish this response. This effect is also believed to be a function of AT_1_R activation within the carotid body. Considering these two extremes, the dosing protocol used in this study should have been adequate to detect any significant physiological outcomes.

We may not have seen a potentiation in chemosensitivity following nocturnal hypoxia because of the time of day the chemoreceptor reflex was measured. Chemoreceptor reflex sensitivity is affected by circadian rhythms independent of changes in metabolic rate (Stephenson et al., 2000). Although basal ventilation remains constant, the chemoreceptor reflex response to hypercapnia is heightened at night and attenuated during the day. This may explain why we did not see a greater difference in the chemoreflex response following exposure to nocturnal hypoxia. Since all participants performed both placebo and treatment arms of this study, diurnal variations in chemoreceptor sensitivity would not have influenced the effect of losartan on ventilatory response to hypercapnia or apnea.

### Conclusion

The current study examined whether AT_1_R blockade attenuates chemoreceptor reflex sensitivity, loop gain, and CSA severity in healthy, young males using a model of nocturnal hypoxia. We found that losartan did not influence chemoreceptor reflex sensitivity, loop gain, or the severity of CSA. Interestingly, we observed a strong relationship between dynamic loop gain measured during sleep and the severity of hypoxia-induced CSA, consistent with the view that the manifest pattern was indeed chemoreflex driven as intended. Overall, our data show that activation of the AT_1_R does not contribute to the chemoreceptor response to hypercapnia including its central and peripheral contributions, before or after a single night of sustained hypoxia in healthy males. It remains feasible that the AT_1_R may contribute to chemoreceptor reflex sensitivity in pathological states such as heart failure, obstructive sleep apnea, or following chronic hypoxia.

## Data Availability Statement

The datasets generated for this study are available on request to the corresponding author.

## Ethics Statement

This study involving human participants was reviewed and approved by Clinical Research Ethics Board, University of British Columbia. The participants provided their written informed consent to participate in this study.

## Author Contributions

CB, RW, SS, and GF: study design. CB, LB, TV, and GF: data collection. CB, LB, SS, and GF: data analysis. All authors: Interpretation, drafting and approval of the manuscript.

## Conflict of Interest

SS has worked as a consultant for Nox Medical, Merck, and Apnimed; he also received grant support from Apnimed and Prosomnus. The remaining authors declare that the research was conducted in the absence of any commercial or financial relationships that could be construed as a potential conflict of interest.

## Footnotes

1 www.random.org
